# Regulation of *Plasmodium* sporozoite motility by formulation components

**DOI:** 10.1186/s12936-019-2794-y

**Published:** 2019-05-02

**Authors:** Clarize M. de Korne, Luuk T. Lageschaar, Matthias N. van Oosterom, Els Baalbergen, Beatrice M. F. Winkel, Severine C. Chevalley-Maurel, Aldrik H. Velders, Blandine M. D. Franke-Fayard, Fijs W. B. van Leeuwen, Meta Roestenberg

**Affiliations:** 1 Department of Parasitology, Leiden University Medical Center, Albinusdreef 2, PO BOX 9600, 2300 RC Leiden, The Netherlands; 2 Interventional Molecular Imaging Laboratory, Department of Radiology, Leiden University Medical Center, Albinusdreef 2, PO BOX 9600, 2300 RC Leiden, The Netherlands; 3 Laboratory of BioNanoTechnology, Axis, Building 118, Bornse Weilanden 9, 6708 WG Wageningen, The Netherlands; 4 Department of Infectious Diseases, Leiden University Medical Center, Albinusdreef 2, PO BOX 9600, 2300 RC Leiden, The Netherlands

**Keywords:** Motility regulation, Live in vitro imaging, *Plasmodium berghei*, Vaccine formulation

## Abstract

**Background:**

The protective efficacy of the most promising malaria whole-parasite based vaccine candidates critically depends on the parasite’s potential to migrate in the human host. Key components of the parasite motility machinery (e.g. adhesive proteins, actin/myosin-based motor, geometrical properties) have been identified, however the regulation of this machinery is an unknown process.

**Methods:**

In vitro microscopic live imaging of parasites in different formulations was performed and analysed, with the quantitative analysis software SMOOT_*In vitro*_, their motility; their adherence capacity, movement pattern and velocity during forward locomotion.

**Results:**

SMOOT_*In vitro*_ enabled the detailed analysis of the regulation of the motility machinery of *Plasmodium berghei* in response to specific (macro)molecules in the formulation. Albumin acted as an essential supplement to induce parasite attachment and movement. Glucose, salts and other whole serum components further increased the attachment rate and regulated the velocity of the movement.

**Conclusions:**

Based on the findings can be concluded that a complex interplay of albumin, glucose and certain salts and amino acids regulates parasite motility. Insights in parasite motility regulation by supplements in solution potentially provide a way to optimize the whole-parasite malaria vaccine formulation.

**Electronic supplementary material:**

The online version of this article (10.1186/s12936-019-2794-y) contains supplementary material, which is available to authorized users.

## Background

In 2017, an estimated 219 million cases of malaria occurred worldwide leading to nearly half a million deaths, which makes malaria the most deadly parasitic disease worldwide (according to the World Health Organization, World Malaria Report 2018). Infection with malaria is initiated when *Plasmodium* parasites are injected into the skin by a probing *Anopheles* mosquito in the highly motile sporozoite (spz) stage [1]. Motility enables spz to exit the skin site, enter the bloodstream and reach and infect the liver, which makes spz motility a target for anti-malarial drugs and vaccines [2]. In addition, the potency of malaria vaccine candidates based on live attenuated spz depends on their potential to migrate in the human host, infect hepatocytes and induce an immune response, which cannot be replicated by dead sporozoites [3]. Nevertheless, the regulation of this migratory behaviour is still an unknown process.

After intradermal deposition, spz rely on their own adhesion capacity and actin/myosin-based motility machinery to migrate. Their adhesion capacity relies on proteins, e.g. the circumsporozoite protein (CSP) and the thrombospondin-related adhesive protein (TRAP), which can form adhesion sites [4, 5]. The establishment of new adhesion sites and the release of the old, enabled by the actin/myosin motor, provides the forward locomotion at a speed linked to the turnover rate [6]. The subsequent direction of this movement is related to the geometrical properties (the crescent shape and the presence of polar rings) of the spz which induce chirality [7, 8]. Spz motility is required to move out of the dermal tissue and to reach the bloodstream. At that point the blood flow will transport spz to the liver where they can invade hepatocytes [9, 10]. As a result, the migration process of spz and possibly thereby also their infectivity is directly influenced by their motility [11, 12]. Although the presence and importance of the elements of the spz motility machinery are confirmed, the regulation of e.g. adhesive protein secretion, actin/myosin motor activity and spz chirality is still poorly understood [9, 13, 14]. Given the importance of spz motility, a better understanding of stimuli that promote and inhibit spz motility is needed [11, 12].

To shed light on spz motility, transgenic spz e.g. *Plasmodium berghei* and *Plasmodium yoelii* expressing fluorescent proteins (e.g. green fluorescent protein; GFP) [15] have been generated for intravital studies [16, 17]. These studies showed that spz display complex motility in their natural environment [18–20], possibly due to tissue morphology and the availability of nutrients in the extracellular matrix. In order to dissect the role of these factors separately, more simplified in vitro models are needed [13, 21]. Studies using 3D in vitro environments revealed that physical confinement plays an important role in regulating the direction and velocity of spz movement [22, 23]. However, when physical confinement is taken out of the equation, the availability of chemical stimuli is revealed as a crucial regulator of spz motility. So far, reports on in vitro spz motility have shown that albumin and calcium act as essential stimuli of spz motility [24–26]. More research is needed to characterize other stimuli of spz motility.

Classically, gliding assays are used as the gold standard for assessing spz motility in vitro [27, 28], but this assay is not ideal for exploring spz motility regulation, because it only provides indirect analysis of spz motility performed through post hoc assessment of spz trails [5, 29]. What is lacking to date, is a quantitative in vitro analysis tool that allows real time detailed spz motility characterization.

In vitro microscopic imaging studies with *Plasmodium berghei*-*mCherry* when augmented with a quantitative analysis tool have the potential to provide a means to assess the regulation of *P. berghei* adhesion and locomotion by formulation composition. To illustrate the potential of this concept, the custom image analysis software called SMOOT_*In vitro*_ (Sporozoite Motility Orienting and Organizing Tool) has been developed and used to study the effect of a systematic variation in formulations: PBS, HBSS and RPMI, enriched with either albumin or whole serum.

## Methods

### Preparation of sporozoites

Mosquitoes were infected by feeding on infected mice (female OF1 mice, 6–7 weeks old; Charles River, Leiden, The Netherlands) as described previously [30]. The animal experiments of this study were performed in accordance with Dutch welfare regulations and were approved by the institutional Animal Ethics Committee of the Leiden University Medical Center (DEC PE.18.005.001). *Plasmodium berghei* spz (*Pb*ANKA-mCherry_hsp70_ + Luc_eef1α_; line RMgm-1320, http://www.pberghei.eu) which contain the fusion gene *mcherry* under control of the strong *hsp70* promoter integrated into the neutral *230p* gene locus (PBANKA_0306000) were obtained by microsurgical dissection of the salivary glands of infected female *Anopheles stephensi* mosquitoes. To allow the spz to mature dissections were performed 21–24 days following infection of the mosquitoes. During this period mosquitoes were kept at a temperature of 21 °C and 80% humidity. The salivary glands were collected and crushed in the different formulations mentioned in the next section. The free spz were counted in a Bürker counting chamber using phase-contrast microscopy. On average, dissection yielded 55 k spz/mosquito. The mosquitoes and the spz were kept on ice (0–4 °C) for a maximum of 3 h until use.

### Sporozoite motility analysis set-up

To investigate which components are needed to induce motility of spz, the mosquitoes were dissected and the obtained salivary glands were crushed in eleven different formulations: (1) Phosphate buffered saline which contains potassium, sodium, chloride and phosphate (PBS; Life Technologies Inc.), (2) Hanks’ balanced salt solution which contains besides the salts present in PBS also glucose, calcium, magnesium, sulphate and bicarbonate (HBSS; Life Technologies Inc.), (3) Roswell Park Memorial Institute medium (RPMI; Life Technologies Inc.) which contains besides the components of HBSS also amino acids and vitamins, (4) PBS enriched with 3.5 mg/ml bovine serum albumin (BSA; Sigma-Aldrich), (5) HBSS + 3.5 mg/ml BSA, (6) RPMI + 3.5 mg/ml BSA, (7) PBS enriched with 10% fetal bovine serum (FBS; Life Technologies Inc.), (8) HBSS + 10% FBS, (9) RPMI + 10% FBS, (10) RPMI without amino acids (Caisson Labs) + 3.5 mg/ml BSA and (11) RPMI without glucose (Life Technologies Inc.) + 3 mg/ml BSA. All eleven formulations had a physiological pH of 7.0–7.5 and a viscosity of < 1.1 cP. For imaging of the spz, 10 µl of the spz solution was pipetted on the cover slip of a confocal dish without any precoating (ø14 mm; MatTek Corporation), covered with another cover slip (ø12 mm; VWR Avantor) and imaged within 45 min. The set-up is schematically depicted in Additional file 1: Fig. S1.

### Confocal imaging

Images of the spz were taken on a Leica TCS (true confocal scanning) SP5 or SP8X WLL (white light laser) microscope (Leica Microsystems, Wetzlar). The spz expressed mCherry which was excited at 587 nm and the emission was collected between 600 and 650 nm. The movies were recorded with a frame rate of 35 frames per minute, 400 frames per movie, three movies per condition. The resolution of the images was set at 1024 × 1024 pixels. For imaging of the spz a 40× objective (Leica HCX PL APO CS 40×/1.25–0.75 na OIL) was used and the experiments were performed at room temperature, unless otherwise stated. The movies were recorded using the Leica software (LAS X version 1.1.0.12420; Leica Microsystems, Wetzlar).

### Movie analysis

Maximum projections of the recorded microscopy movies were generated using Fiji software [31]. The movies were further processed using SMOOT_*In vitro*_, an in-house developed graphical user interface (GUI), written in the MATLAB programming environment (version r2017b, The MathWorks Inc.). Via SMOOT_*In vitro*_ analysis the spz could be segmented per movie frame, based on their fluorescence signal intensity, size and crescent shape by applying a binary threshold, a median filter, skeleton adjustment and spot removal. Colliding spz and spz circling on the edge of the field of view were excluded, since the locations of these spz on the consecutive frames cannot be stitched together in a reliable way. The value of these segmentation parameters can be set in the GUI. The direct visual feedback provided by the GUI during the setting of the parameters was used to find the optimal segmentation settings. Over time the median pixel locations of segmented spz visible on different frames could be connected into full spz tracks, which were subsequently separated in segments based on changes in movement pattern. The movement pattern of the spz was classified per segment as floating, stationary or circling. The velocity and turn angle at frame level, the turn direction (clockwise (CW) or counter-CW (CCW)) at segment level and the length of the travelled path (expressed as number of frames and displacement per frame) at track level of the circling spz were determined. On average ~ 100 spz were analysed per condition.

### Statistics

Statistical analysis consisted of the One-way ANOVA, the Mann–Whitney U test and the independent sample *t* test depending on the nature of the variables tested (pre-test of normality: Shapiro–Wilk test; pre-test of variance homogeneity: Levene-test). p-values < 0.05 were considered significant. All statistical tests were performed by SPSS Statistics (version 23; IBM Nederland B.V.).

## Results

### Sporozoite movement patterns

**Fig. 1:**
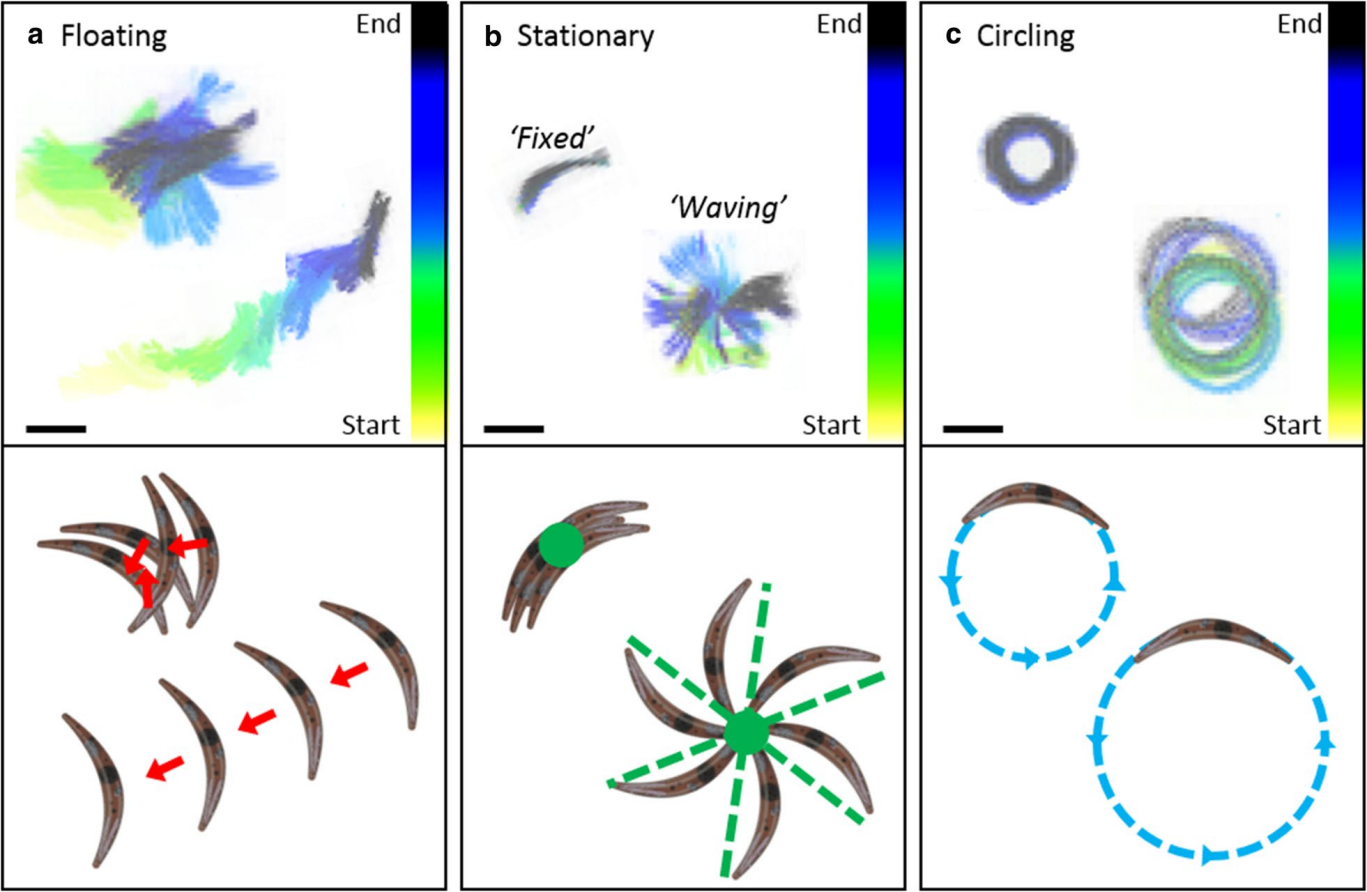
Spz movement patterns. Maximum projections of movies showing the different movement patterns of spz on a glass surface: spz floated within the solution (**a**), spz fully attached to the surface “fixed” or with one tip attached to the surface “waving” (**b**), and spz were turning in circles with varying diameters (**c**). The moving patterns seen at the maximum projections are also schematically depicted. Scalebar: 10 µm

SMOOT_*In vitro*_ was able to discern three main spz movement patterns in the microscopic spz movies: floating (red), stationary (green) and circling (blue; Fig. 1). The floating spz could not attach to the surface and as such displayed random movement based on fluidics (Fig. 1a). Overall, 3% of the total number of spz imaged were considered to be stationary, either by full length or partial surface attachment. Uniquely the latter results in a “waving” phenomenon (47% of stationary spz, Fig. 1b). Furthermore, motile spz were observed which attached to the surface and turned in circles (Fig. 1c). The majority of circling spz moved in a CCW direction, only 2% of them turned CW. Whilst the CCW movement was often sustained during the whole movie, CW-turning spz only turned on average 1.5 circles (range 1–7 circles) before detaching from the surface. The different formulations did not influence the ratio between CCW and CW movement (Additional file 2: Table S1).

### Movement pattern quantification

**Fig. 2:**
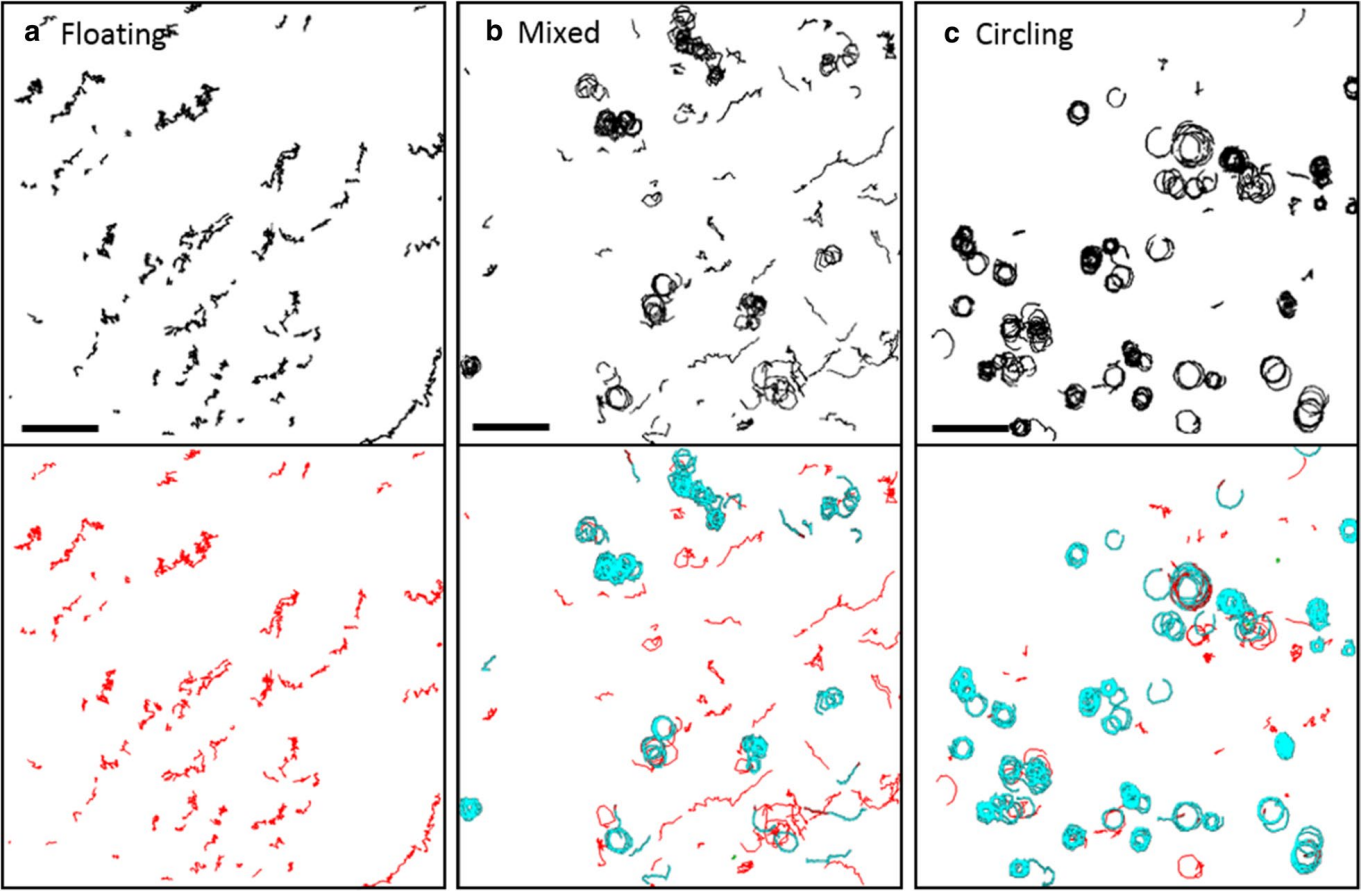
Effect of formulation on spz movement. Overview of tracks of spz under three different conditions: without FBS or BSA only floating spz were seen (**a**), HBSS + BSA resulted in a mix of both floating and circling spz (**b**) and other conditions with BSA and FBS showed primarily circling spz. The tracks of the lower row are color-coded; floating tracks are depicted in red, circling tracks are depicted in blue and stationary tracks are depicted in green. Scalebar: 50 μm

Using automated SMOOT_*In vitro*_ movement pattern classification, a matrix of nine different formulations (f1–9) was studied: PBS, HBSS and RPMI (f1–3), which were enriched with either 3.5 mg/ml purified BSA (f4–6) or 10% FBS which contains amongst other things ~ 3.5 mg/ml BSA (f6–9). Changes in the formulation yielded a direct effect on the spz movement patterns. Typically, in formulations without BSA or FBS (f1–3) and also in the condition PBS + BSA (f4), most spz were floating. An example of such an effect is shown in Fig. 2a. In the formulation HBSS + BSA both floating and circling spz were seen (f5, Fig. 2b), whereas in the other conditions with BSA and FBS the majority of spz were circling (f6–9, Fig. 2c).

**Fig. 3:**
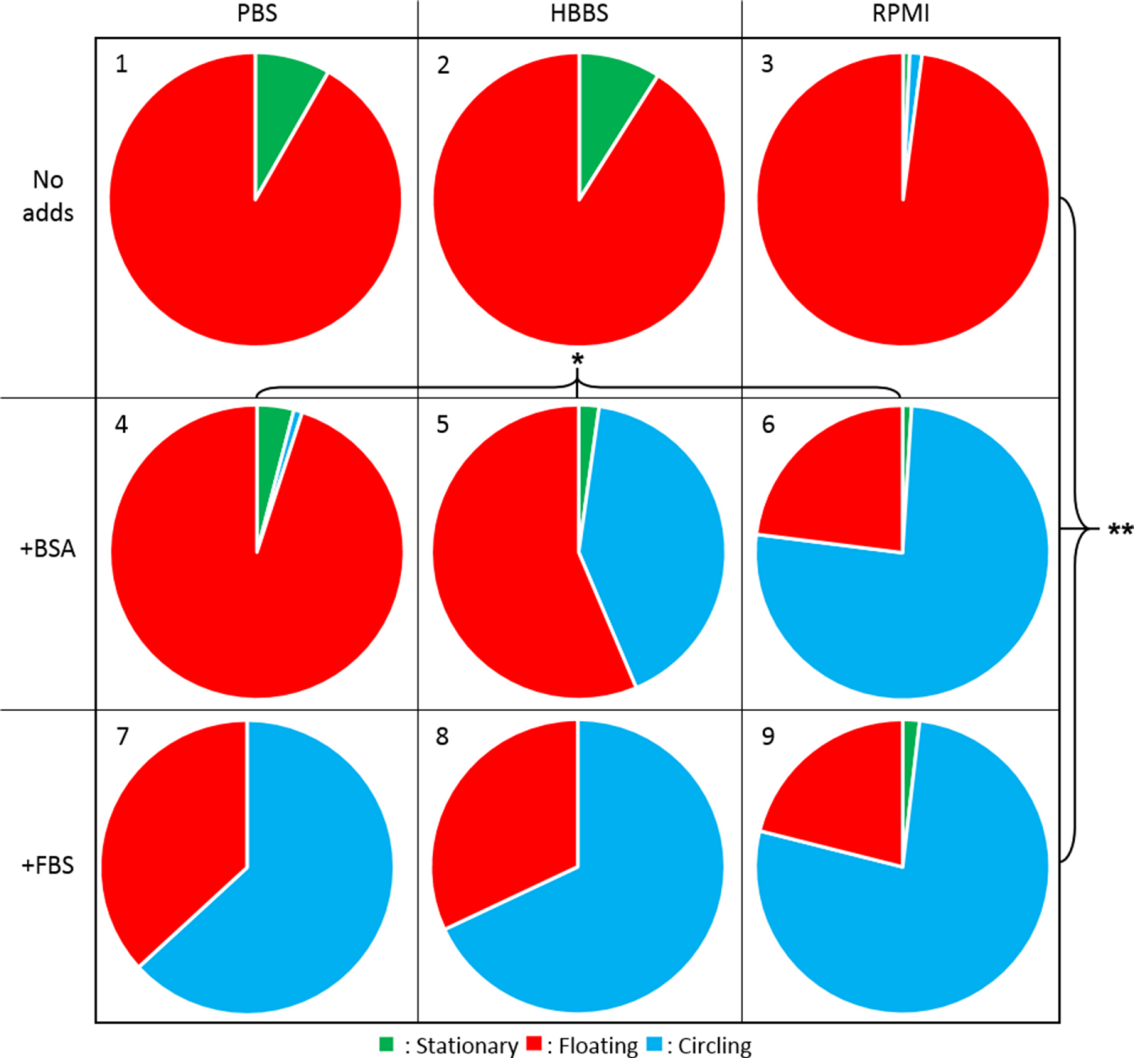
Movement pattern distribution. The movement pattern distribution for spz in the 9 different solutions. More spz were circling in the solutions enriched with BSA (4–6) and FBS (7–9) compared to the non-enriched solutions (1–3) (p = 0.007; One-way ANOVA). In RPMI enriched with BSA or FBS (6, 9), the spz circled more compared to PBS (4, 7) and HBSS (5, 8) enriched with BSA or FBS (p = 0.014; One-way ANOVA). No significant difference was found between RPMI enriched with BSA or FBS (p = 0.823; independent sample t-test). *p < 0.05, **p < 0.01

Quantitative analysis confirmed that the movement behaviour of spz was significantly different between the conditions with and without BSA and FBS (p = 0.007; One-way ANOVA; Fig. 3). Without FBS or BSA, 92% of the spz were floating, only a 7% minority of the spz could attach to the surface and were stationary (Fig. 3: 1–3). These stationary spz were either fully attached (41%) or were waving (59%). Interestingly, waving spz were observed significantly more in the formulations which did not induce circling (Fig. 3: 1–4) than in the formulations which could (Fig. 3: 5–9; resp. 94% and 6%, p = 0.026; independent sample t = test), suggesting that waving and circling are mutually exclusive. It thus seems that spz which are unable to circle can (partially) adhere, but then cannot progress to the next movement phase and as a consequence start waving.

**Fig. 4:**
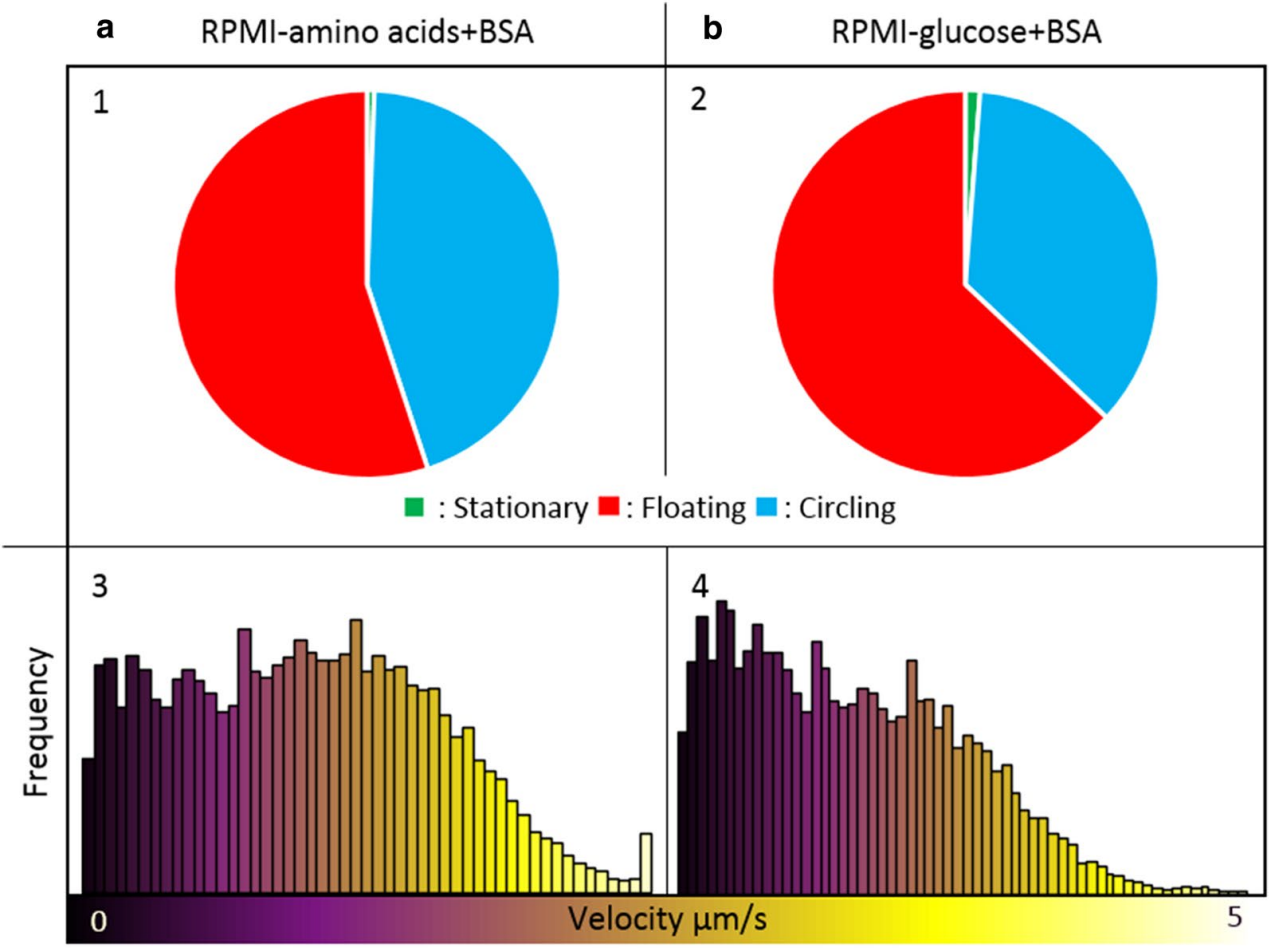
Effect of amino acids and glucose. The movement pattern distribution for spz in RPMI without amino acids (**a1**) and without glucose (**b2**) both enriched with BSA and the velocity distribution for spz in RPMI without amino acids (**a3**) and without glucose (**b4**) both enriched with BSA are plotted. Under the condition without amino acids and without the glucose, the spz circled on average with a velocity of 2.0 µm/s and 1.7 µm/s respectively

Conforming previous data, BSA proved to be an essential supplement [24] since HBSS and RPMI enriched with 3.5 mg/ml BSA induced circling (Fig. 3: 5–6). However, BSA was not the only essential supplement since PBS + BSA was not able to induce circling (Fig. 3: 4). The following trend was seen for the three formulations enriched with BSA: PBS, a phosphate-buffered sodium chloride solution, did not induce circling, HBSS which contains additional ions (e.g. calcium, magnesium and bicarbonate) and glucose induced 41% circling spz and RPMI, which contains salts, glucose, amino acids and vitamins increased the percentage of circling spz to 76% (Fig. 3: 4–6). This trend revealed that besides BSA, also salts, glucose and certain amino acids and/or vitamins were essential supplements to induce attachment and circling. The role of glucose and amino acids was dissected in more detail by removing these components from the RPMI + BSA formulation (f6). Both depletion of amino acids and glucose resulted in a significant decrease of spz adherence capacity: 22% of the spz were floating in RPMI + BSA, 55% of the spz were floating in the solution without amino acids (Fig. 4: 1; p = 0.002; independent sample t = test) and 63% of the spz were floating in the solution without glucose (Fig. 4: 2; p = 0.005; independent sample t = test).

FBS contains, in addition to its major component BSA (~ 35 mg/ml), many other components like salts, glucose, vitamins and cholesterol. Enrichment of any of the formulations with FBS induced circling of 60–80% of the spz. Significantly more spz were circling in the presence of FBS compared to BSA when it was added to PBS or HBSS (Fig. 3: 4, 5, 7, 8; p = 0.005; independent sample t-test). In contrast, addition of FBS instead of BSA to RPMI did not further increase the amount of circling spz (Fig. 3: 6, 9; p = 0.816; independent sample t-test). Thus, the supplements present in FBS enhanced the percentage of circling spz, however they did not add value on top of RPMI + BSA: albumin, salts, glucose, amino acids and vitamins.

### Sporozoite velocity during circling

**Fig. 5:**
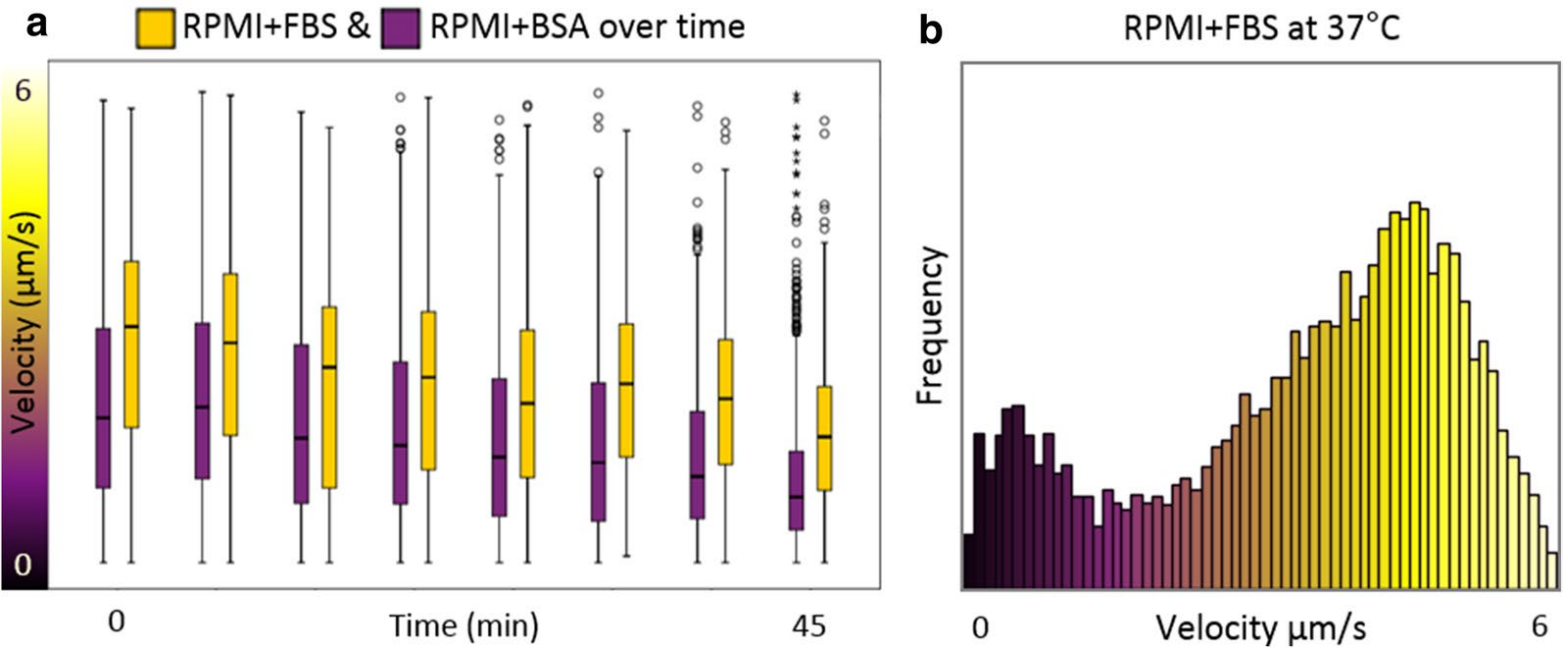
Effect of time and temperature on spz velocity. **a** The velocity of spz in RPMI enriched with FBS (depicted in yellow) or BSA (depicted in purple) is plotted over time. **b** The velocity distribution of the spz circling on a glass surface in RPMI enriched with FBS at 37 °C. On average the spz circled with a velocity of 3.8 µm/s

**Fig. 6:**
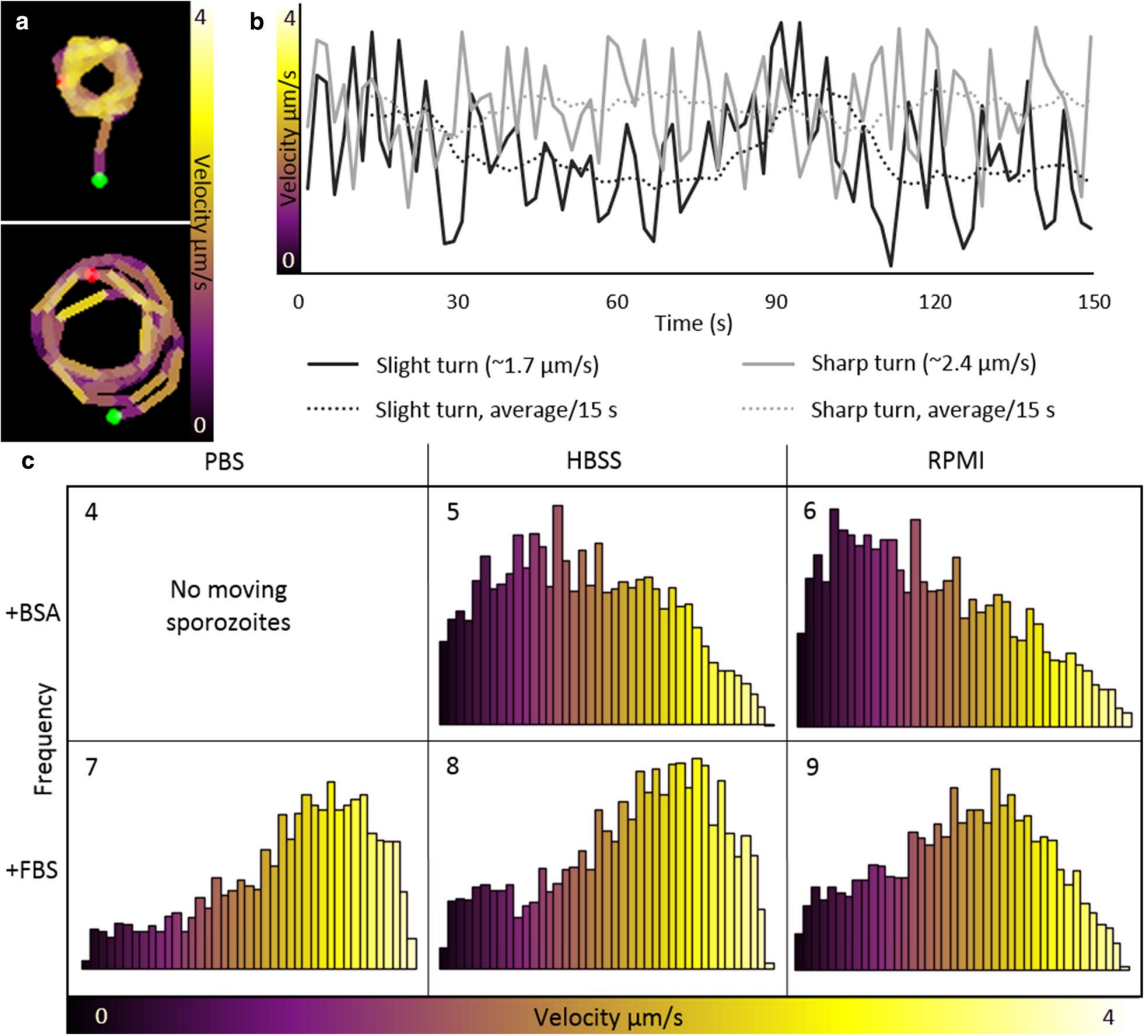
Spz velocity during circling. **a** Velocity heatmaps of a sharp (top) and slight turn (bottom). The start of the tracks is marked with a green dot and the end with a red dot. The velocity of the spz fluctuated along the tracks. **b** The velocity of the spz tracks depicted in A are plotted over time, including a line for the average velocity per 15 s. **c** The velocity distribution of the spz circling on a glass surface in the different formulations. In PBS enriched with BSA, the spz did not circle. In HBSS and RPMI enriched with BSA, the spz circled on average with a velocity of 1.6 µm/s. In the solutions enriched with FBS, the spz circled faster, on average: 2.5 µm/s (p = 0.002; Mann–Whitney U test). Although the median velocity differed between the solutions enriched with BSA and FBS, the velocity ranges were the same (p = 0.796; Mann–Whitney U test)

When studying the velocity of spz in the five different formulations that induced circling (Fig. 3: 5–9), overall, spz displayed circular movement with a median velocity of 2.0 ± 1.0 µm/s. When comparing the different formulations, the median velocity was 1.4 and 1.7 µm/s for spz supplemented with BSA (HBSS and RPMI respectively), whereas spz supplemented with FBS exhibited higher velocities; 2.1, 2.5 and 2.7 µm/s (RPMI, HBSS and PBS respectively). This velocity difference persisted over time (Fig. 5a). Besides the components available in solution, also temperature influenced the velocity of the spz. The velocity of spz in RPMI supplemented with FBS nearly doubled after increasing the temperature from room temperature to 37 °C (Fig. 5b; median velocity: 3.8 µm/s at 37 °C versus 2.1 μm/s at room temperature). The spz did not move at a constant speed, their velocity fluctuated along the track, which was visualized by colour-coding (Fig. 6a, b). Yellow sections, corresponding to a high velocity, alternated with purple sections, corresponding to a lower velocity. Given these velocity fluctuations along the track, the velocity of the spz was analysed per frame instead of analysing average track velocities.

Although the range of velocities was similar for spz in the five different solutions (~ 1% of the spz could reach a velocity of 4.0 µm/s under all conditions), between these formulations striking variations could be observed in the distribution of the velocities measured (Fig. 6c: 5–9). Clearly the velocity of the spz was not normally distributed (p < 0.001; Shapiro–Wilk test). Two different trends were seen. First, the spz in medium enriched with FBS moved significantly faster than the spz enriched with BSA (p = 0.002; Mann–Whitney U test). Second, within the three formulations enriched with FBS, spz in PBS moved faster than spz in RPMI (p = 0.050; Mann–Whitney U test). Although less spz started circling in RPMI without amino acids compared to complete RPMI, for the spz which started circling a shift to higher velocities was observed (Fig. 4: 3). Interestingly, this shift was related to a significant decrease in the diameter of the circles (Additional file 3: Fig. S2; 12.3 µm in complete RPMI, 10.3 µm in RPMI without amino acids; p < 0.001; independent sample t-test). Hence, the availability of specific (macro)molecules in the formulation seemed to determine not only spz adherence and their capacity of forward locomotion but also the velocity at which spz were able to move.

## Discussion

The availability of a dedicated analysis tool (SMOOT_*In vitro*_) enabled a quantitative analysis of spz motility by describing their (1) attachment rate, (2) movement pattern distribution and (3) velocity distribution. Because SMOOT_*In vitro*_ enabled to tease apart spz motility in tracks, segments and frames, the different motility parameters could be individually dissected and subtle differences could be quantified. In this way has been confirmed that albumin is essential to induce spz attachment and movement. Glucose, salts, amino acids and vitamins, components in RPMI, further increased the attachment rate and the percentage of moving spz, whereas the supplements present in whole serum regulated the velocity of the spz movement. Combined, these findings indicate that the regulation of spz motility is a complex interplay of spz with different macromolecules and salts.

The three in vitro movement patterns, floating, stationary (fully attached and waving) and circling (> 95% CCW), which were observed for *P. berghei* are in line with previous reports [8, 28, 32]. These same reports have also shown that spz could only sustain CW circling for a short period of time. In this study, SMOOT_*In vitro*_ facilitated the analysis of the circular movement patterns at a level of detail beyond what has previously been performed [25, 33]. Particularly, the image processing tool enabled the assessment of unique but subtle alterations in spz velocity along the tracks, which might reflect the turnover rate of spz adhesion sites [6]. The remarkable differences in velocity distribution between the formulations suggested that this level of detail is needed to do justice to the complex interplay between the available (macro)molecules and the spz motility machinery. Since SMOOT_*In vitro*_ allowed for detailed analysis of spz motility at frame level, the tool can be used to further study the action of (novel) motility inhibiting drugs at different concentrations. Potentially, motility analysis with SMOOT_*In vitro*_ could be performed as an additional assay to previously described high-throughput screening methods [2].

Spz attachment and movement are two distinguishable, but likely related steps in spz motility. Both appear to be initiated by external stimuli transferred by internal signalling cascades. Hegge et al. have described spz adherence and movement as a four step procedure [34]: step 1 is the initial adhesion with one tip, step 2 the formation of secondary adhesion site, step 3 full body attachment, step 4 the initiation of gliding. According to literature, step 2–4 of this adhesion model should be dependent on the secretion of adhesive proteins triggered by albumin and the turnover of these adhesion sites by the actin/myosin-based molecular motor [6, 24, 35]. The latter requires a considerable sustained internal energy production. In accordance with the proposed model, without the presence of albumin and glucose only a few spz (7%) could achieve full body attachment (step 3) and none of the spz started gliding (step 4). Albumin strongly promoted full body attachment and glucose further increased it (step 3), however in these conditions also gliding was initiated (step 4). In conclusion, our data supports the notion that spz attachment and movement are related steps in spz motility.

Interestingly, albumin and glucose do not seem to be the sole supplements regulating spz motility. In addition, supplements present in RPMI (which, besides salts and glucose also contains amino acids and vitamins) act as stimuli for spz attachment and subsequent moving. As spz can also use glutamate or glutamine to enter the Krebs cycle to produce energy and the availability of these compounds influences spz motility [10, 33], this could explain why depletion of the amino acid content of RPMI decreased the percentage of attached and moving spz compared to PBS and HBSS.

The available (macro)molecules did not only regulate the occurrence of spz adherence and moving, but also the velocity of their forward locomotion. Besides attachment to the surface via adhesive proteins, also detachment by the turnover of the adhesion sites is essential to allow the spz to move forwards at a certain speed [6, 36]. This attachment–detachment process could have caused the fluctuating velocity we measured at frame level. Strikingly, whole serum induced a distribution shift to higher velocities of spz compared to albumin without changing the maximum reached velocity. It is still unclear which of the > 30 components that whole serum consists of is responsible for this shift. Nevertheless, the change in velocity distribution induced by whole serum reveals that besides the attachment rate and the initiation of movement, the velocity of spz movement is clearly also regulated by the formulation composition.

The comparison between the attachment rate, movement pattern distribution and velocity distribution induced by the different formulations suggested two different trends: (1) whole serum both increased the attachment rate, the percentage of circling spz and their velocity compared to albumin and (2) RPMI increased the attachment rate and the percentage of circling spz, but decreased their velocity compared to PBS and HBSS. Combined these trends indicate that spz motility is dependent on regulators inducing the right balance between strong attachment and fast detachment. The SMOOT_*In vitro*_ analysis of the effect of different potential stimuli and inhibitors has the potential to provide insight in the biological mechanisms behind spz motility. A recently developed fluorescent labelling approach for non-fluorescent parasites provides the opportunity to study the motility of spz in vaccine preparations or isolated in the field [37]. Potentially, SMOOT_*In vitro*_ can be adapted to study the motility of malaria parasites in other developmental stages (e.g. ookinetes) if the segmentation and stitching parameters would be adjusted and validated to select the parasite.

Despite the regulating role of the different supplements on spz motility, PfSPZ vaccines currently are formulated in PBS with albumin only [38]. It is not yet clear to what extent the observed in vitro formulation effects can impact the in vivo situation where proteins, glucose, salts and amino acids are in supply from the surrounding blood/tissue. However, this should be the subject of further research, as the addition of glucose, salts and amino acids will potentially improve spz motility after deposition in the human skin and thereby may enhance the potency of whole-parasite malaria vaccines.

## Conclusions

This initial in vitro study indicated that (macro)molecules effect spz motility by acting as regulators of adherence to the surface, induction of forward locomotion and the velocity of this movement. SMOOT_*In vitro*_ allowed for a quantitative assessment of the different conditions studied and with that helped isolate the supplements that were key for spz motility. This spz motility analysis tool can be of future use to gain insight in the mechanisms behind spz migratory behaviour, to determine the most optimal vaccine formulation, to study the effect of radiation and chemical attenuation of spz and to screen potential spz motility inhibiting drugs.

## Additional files

**Additional file 1: Figure S1.** Spz motility analysis tool. A) The *Plasmodium berghei* spz were obtained by manual dissection and crushing of the salivary glands of infected *Anopheles stephensi* mosquitoes. B) The behaviour of spz in different types of media was studied. C) The spz were imaged by confocal microscopy and analyzed using the software SMOOT.

**Additional file 2: Table S1.** (Counter) clockwise movement. A) Distribution of clockwise (CW) and counter-clockwise (CCW) turning spz for the pooled dataset. B) Distribution of CW and CCW turning spz per condition.

**Additional file 3: Figure S2.** Sporozoites circling diameter. The average diameter of the circles of turning sporozoites in RPMI and RPMI without glucose (both enriched with BSA) were 12.3 µm and 12.2 µm respectively. The average diameter of the circles of turning sporozoites in RPMI without amino acids and enriched with BSA was significantly smaller: 10.3 µm (p < 0.001; independent sample t-test).

## Abbreviations

Spz: sporozoites
CSP: circumsporozoite protein
TRAP: thrombospondin-related adhesive protein
GFP: green fluorescent protein
RPMI medium: Roswell Park Memorial Institute medium
FBS: fetal bovine serum
PBS: phosphate buffered saline
HBSS: Hanks’ balanced salt solution
BSA: bovine serum albumin
SMOOT: Sporozoite Motility Orienting and Organizing Tool
(C)CW: (counter-)clockwise
