# Supplementary figures and images for: Regulation of Plasmodium sporozoite motility by formulation components
Source: Malar J. 2019 May 2;18:155. doi: 10.1186/s12936-019-2794-y (PMC6498664; doi:10.1186/s12936-019-2794-y)

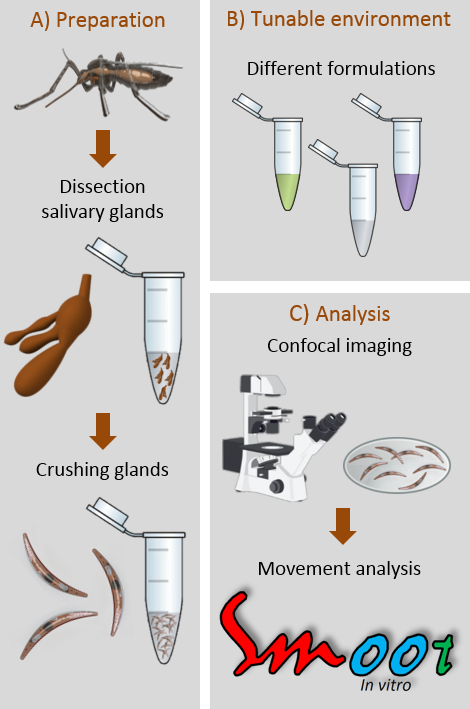

Supplement: Supplementary file 1 — Additional file 1: Figure S1. Spz motility analysis tool. A) The Plasmodium berghei spz were obtained by manual dissection and crushing of the salivary glands of infected Anopheles stephensi mosquitoes. B) The behaviour of spz in different types of media was studied. C) The spz were imaged by confocal microscopy and analyzed using the software SMOOT. [file 12936_2019_2794_MOESM1_ESM.tif]

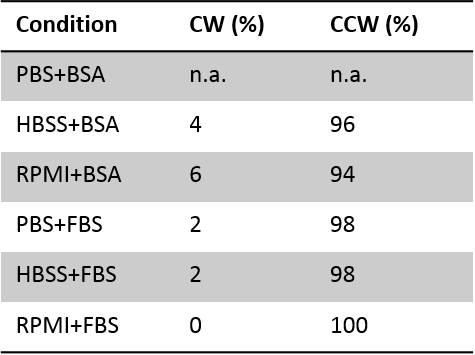

Supplement: Supplementary file 2 — Additional file 2: Table S1. (Counter) clockwise movement. A) Distribution of clockwise (CW) and counter-clockwise (CCW) turning spz for the pooled dataset. B) Distribution of CW and CCW turning spz per condition. [file 12936_2019_2794_MOESM2_ESM.png]

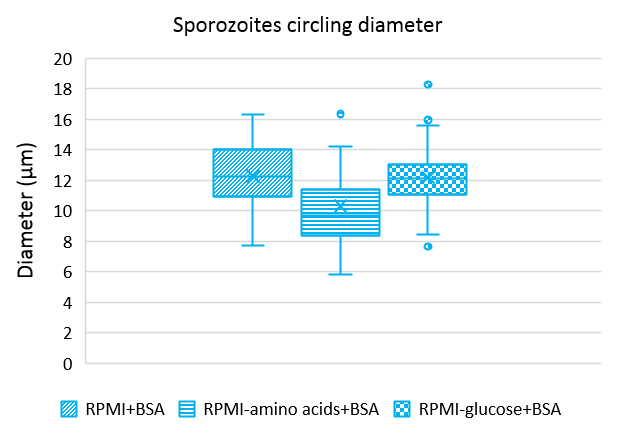

Supplement: Supplementary file 3 — Additional file 3: Figure S2. Sporozoites circling diameter. The average diameter of the circles of turning sporozoites in RPMI and RPMI without glucose (both enriched with BSA) were 12.3 µm and 12.2 µm respectively. The average diameter of the circles of turning sporozoites in RPMI without amino acids and enriched with BSA was significantly smaller: 10.3 µm (p < 0.001; independent sample t-test). [file 12936_2019_2794_MOESM3_ESM.png]
